# West Nile virus in overwintering mosquitoes, central Europe

**DOI:** 10.1186/s13071-017-2399-7

**Published:** 2017-10-02

**Authors:** Ivo Rudolf, Lenka Betášová, Hana Blažejová, Kristýna Venclíková, Petra Straková, Oldřich Šebesta, Jan Mendel, Tamás Bakonyi, Francis Schaffner, Norbert Nowotny, Zdeněk Hubálek

**Affiliations:** 1 Institute of Vertebrate Biology, Czech Academy of Sciences, v.v.i, Brno, Czech Republic; 2 Department of Experimental Biology, Masaryk University, Brno, Czech Republic; 3 Institute of Macromolecular Chemistry, v.v.i., Czech Academy of Sciences, Praha, Czech Republic; 4 Department of Microbiology and Infectious Diseases, University of Veterinary Medicine, Budapest, Hungary; 5 Institute of Virology, University of Veterinary Medicine, Vienna, Austria; 6 Francis Schaffner Consultancy, Riehen, Switzerland; 7 National Centre for Vector Entomology, Institute of Parasitology, Vetsuisse Faculty, University of Zurich, Zurich, Switzerland; 8 Department of Basic Medical Sciences, College of Medicine, Mohammed Bin Rashid University of Medicine and Health Sciences, Dubai Healthcare City, Dubai, United Arab Emirates

**Keywords:** West Nile fever, West Nile virus, Flavivirus, Hibernation, Overwintering, *Culex pipiens*, *Anopheles maculipennis*, *Culiseta annulata*, Czech Republic

## Abstract

**Background:**

West Nile virus (WNV) is currently the most important mosquito-borne pathogen spreading in Europe. Data on overwintering of WNV in mosquitoes are crucial for understanding WNV circulation in Europe; nonetheless, such data were not available so far.

**Results:**

A total of 28,287 hibernating mosquitoes [27,872 *Culex pipiens*, 73 *Anopheles maculipennis* (*sensu* *lato*), and 342 *Culiseta annulata*], caught in February or March between 2011 and 2017 in a WNV-endemic region of South Moravia, Czech Republic, were screened for the presence of WNV RNA. No WNV positive pools were found from 2011 to 2016, while lineage 2 WNV RNA was detected in three pools of *Culex pipens* mosquitoes collected in 2017 at two study sites.

**Conclusions:**

To the best of our knowledge, this is the first record of WNV RNA in overwintering mosquitoes in Europe. The data support the hypothesis of WNV persistence in mosquitoes throughout the winter season in Europe.

## Background

West Nile virus (WNV) is a mosquito-borne virus (genus *Flavivirus*; family *Flaviviridae*) with nearly cosmopolitan distribution [1]. In nature, it circulates between birds (as amplifying hosts) and bird-feeding mosquitoes, in Europe predominantly *Culex pipiens* [2, 3]. Humans and horses are considered accidental dead-end hosts. In 2004 a neuroinvasive lineage 2 WNV (WNV-2) was discovered for the first time in Europe, i.e. in south-eastern Hungary [4]. Since 2008, an unexpected explosive spread of this WNV-2 resulted in several hundreds of human and animal neuroinvasive cases in Hungary [5], Austria [5, 6], Greece [7, 8], Serbia [9], and Italy [10]. In the Czech Republic, four identical strains of WNV-2 were isolated from *Cx. modestus* mosquitoes collected in reed beds at South-Moravian fishponds in 2013 [11].

Overwintering of the introduced WNV-2 in Europe was assumed (e.g. [4, 5]), yet larger systematic studies which specifically focus on the persistence of WNV in overwintering mosquitoes are lacking in a European WNV endemic area. However, answering this question is crucial for the understanding of long-term persistence of WNV and the WNV epidemiology in general in Europe, especially in northern endemic regions. In contrast, in the United States, several such investigations were carried out, and WNV was occasionally detected in diapausing *Cx. pipiens*, albeit at very low rates [12–15].

A long-term (seven-year) study was therefore undertaken to examine the prevalence of WNV in overwintering populations of mosquitoes collected from hibernacula located in a WNV endemic region in South Moravia. We performed molecular screening of female *Cx. pipiens*, *Anopheles maculipennis* (*s*.*l*.) and *Culiseta annulata* mosquitoes (they overwinter as nulliparous females) for WNV to evaluate the hypothesis of hibernation of WNV in overwintering mosquitoes in central Europe.

## Methods

### Study sites, mosquito collection and identification

Overwintering female mosquitoes were collected by battery-operated aspirators (Hausherr’s Machine Works, Toms River, N.J. 08753, USA) from walls and ceilings of cellars including wine cellars, pensions, a water tower and a castle nearby WNV positive study sites [11]. Mosquito collections were carried out at localities Sedlec, Lednice, Hlohovec, Valtice and Břeclav in South Moravia always in February or March 2011 through 2017 (Table 1). Captured mosquitoes were transported in closed and chilled containers to the laboratory where they were identified on chilled tables under a stereomicroscope, using the determination key of Becker et al. [16] and then stored for further processing in freezers at -60 °C. Mosquitoes of the species *Cx. pipiens* were not further analysed to discriminate between f. *pipiens* and f. *molestus*.

**Table 1:** Study sites and number of overwintering mosquitoes collected in the Czech Republic, 2011 to 2017

Study site	2011			2012			2013			2014			2015			2016			2017		
	*C.p.*	*A.m.*	*C.a.*	*C.p.*	*A.m.*	*C.a.*	*C.p.*	*A.m.*	*C.a.*	*C.p.*	*A.m.*	*C.a.*	*C.p.*	*A.m.*	*C.a.*	*C.p.*	*A.m.*	*C.a.*	*C.p.*	*A.m.*	*C.a.*
Sedlec	2250	0	0	1471	1	3	279	0	1	1132	0	4	1424	0	1	62	0	0	261	0	0
Lednice	3714	7	175	1032	2	23	288	0	5	859	2	11	5187	11	44	446	7	1	732	15	6
Hlohovec	nd	nd	nd	nd	nd	nd	1265	0	0	876	0	39	1441	0	6	528	0	1	166	0	0
Břeclav	1252	26	2	160	0	2	nd	nd	nd	198	1	17	2730	1	1	0	0	0	102	0	0
Valtice	0	0	0	0	0	0	0	0	0	0	0	0	0	0	0	17	0	0	nd	nd	nd
Subtotal	7216	33	177	2663	3	28	1832	0	6	3065	3	71	10,782	12	52	1053	7	2	1261	15	6
Total	7426			2694			1838			3139			10,846			1062			1282		

*Abbreviations*: *C.p.*, *Culex pipiens*, *A.m.*, *Anopheles maculipennis* (*sensu lato*), *C.a.*, *Culiseta annulata*, *nd* not done

### Mosquito homogenization, RNA extraction, PCR analysis and sequencing

Mosquito pools of usually 50 (1 to 100) females sorted by species, year and locality were prepared and homogenised in 1.5 to 2.0 ml of cooled phosphate-buffered saline pH 7.4 with 0.4% bovine serum albumin (fraction V) and antibiotics. RNA was extracted from 150 μl of cooled mosquito homogenates by using the QIAamp Viral RNA Mini Kit (Qiagen, Hilden, Germany), according to the manufacturer’s instructions. The mosquito homogenates were tested by conventional reverse transcription-polymerase chain reaction (RT-PCR) for flaviviral RNA by using the protocol published by Scaramozzino et al. [17], and the Qiagen OneStep RT-PCR Kit (Qiagen) [18, 19]. Sequencing of PCR products and bioinformatic analyses were performed according to a previous study [20].

## Results

A total of 27,872 female overwintering *Cx. pipiens* in 573 pools, 73 *An. maculipennis* (*s*.*l*.) in 15 pools, and 342 *Cs. annulata* in 28 pools collected between 2011 and 2017 were tested for the presence of flavivirus RNA. All mosquito pools from 2011 to 2016 were negative, while in three *Cx. pipiens* pools from 2017 WNV nucleic acid was demonstrated: in specimen 17-06 from Hlohovec collected on 27th February and in two samples from Lednice collected on 21st February (nos. 17-12 and 17-18). Sequencing of partial NS5 fragments (265 nt) of all three strains revealed the closest similarity to WNV strain 13-104 circulating in the area (97% nucleotide identity) [11]. All three sequenced strains were identical to each other. A representative sequence was deposited in the GenBank database under the accession number MF162729. None of the pools was positive for Usutu virus, another important flavivirus which circulates in the region since 2012 [21] and causes epizootics in wild and domestic birds in Europe.

## Discussion

WNV surveillance studies carried out periodically from 2006 through 2017 within the framework of European and national research projects comprising serological surveys of bird, horse and human populations for WNV neutralising antibodies as well as attempts to detect WNV in mosquitoes, horses and humans demonstrated limited WNV activity in the region [22, 23]. The only exception was repeated detection of WNV-2 in *Cx. modestus* and *Cx. pipiens* mosquitoes in 2013 (minimum prevalence rate 0.012%), 2015 (0.099%) and 2016 (0.056%), respectively ([11, 20], unpublished data).

Regarding overwintering mosquitoes, one strain of WNV (strain 99-222, type Rabensburg) was isolated from *Cx. pipiens* females collected in a mini fortress at Lanžhot on 13 October 1999, i.e. from mosquitoes commencing to overwinter [24]. Similarly in Lower Austria, close to places with diagnosed WNV-2 associated bird mortalities, three pools of adult female *Cx. pipiens* mosquitoes caught during October 2008 in cellars proved positive for WNV-2 nucleic acid [5]. Vertical transmission of WNV was confirmed by detecting WNV-2 nucleic acid in one pool of 15 *Cx. pipiens* pupae as well as in a pool of two *Cx. pipiens* egg rafts during a small-scale entomological survey carried out close to the home of a WNV-positive blood donor in Vienna [25].

Given the low WNV-2 endemicity in the Czech Republic until 2013 it is understandable that, at least during the winter periods 2011 to 2013, no WNV-positive hibernating mosquitoes were found. Persistent WNV infections in certain bird species (e.g. as recently demonstrated in an exotic avian species in human care [26]) may to a lesser extent also contribute to the circulation of the virus, either through mosquitoes or oral transmission to predatory birds. Nonetheless, the classical bird-mosquito cycle including vertical transmission in mosquitoes is likely to be the predominant biological cycle of WNV maintenance and transmission. It is worth mentioning that some individuals of the examined pools of *Cx. pipiens* mosquitoes might belong to f. *molestus*, which is characterized by autogeny, stenogamy, anthropophily and facultative diapause [16]. Several publications (e.g. [5]) provide clear-cut genetic evidence that WNV strains, once introduced to central Europe, are maintained there and disperse to neighbouring regions.

## Conclusions

For the first time, WNV was detected in overwintering mosquitoes in Europe. This finding supports the hypothesis of WNV persistence in mosquitoes throughout the winter season in Europe. As a consequence, it can be presumed that lineage 2 WNV infection in Europe is sustained by the long-term persistence of the virus in mosquitoes followed by vertical transmission and by the maintenance of the mosquito-bird transmission cycle, without the necessity of virus re-introductions. However, further studies are needed to analyse in-depth mode of circulation, overwintering and vertical transmission of WNV in central Europe by monitoring overwintering mosquito species, resident bird and horse populations as well as humans.
